# Combining intramuscular and intranasal homologous prime-boost with a chimpanzee adenovirus-based COVID-19 vaccine elicits potent humoral and cellular immune responses in mice

**DOI:** 10.1080/22221751.2022.2097479

**Published:** 2022-07-27

**Authors:** Xingxing Li, Ling Wang, Jingjing Liu, Enyue Fang, Xiaohui Liu, Qinhua Peng, Zelun Zhang, Miao Li, Xinyu Liu, Xiaohong Wu, Danhua Zhao, Lihong Yang, Jia Li, Shouchun Cao, Yanqiu Huang, Leitai Shi, Hongshan Xu, Yunpeng Wang, Yue Suo, Guangzhi Yue, Jianhui Nie, Weijin Huang, Wenjuan Li, Yuhua Li

**Affiliations:** a Department of Arboviral Vaccine, National Institutes for Food and Drug Control, Beijing, People’s Republic of China; b State Key Laboratory of Biotherapy and Cancer Center, West China Hospital, Sichuan University, and Collaborative Innovation Center for Biotherapy, Chengdu, People’s Republic of China; c Department of HIV/AIDS and Sex-transmitted Virus Vaccines, National Institutes for Food and Drug Control, Beijing, People’s Republic of China

**Keywords:** Adenovirus-vectored vaccine, ChAdTS-S, intramuscular, intranasal, SARS-CoV-2

## Abstract

The efficacy of many coronavirus disease 2019 (COVID-19) vaccines has been shown to decrease to varying extents against new severe acute respiratory syndrome coronavirus 2 variants, which are responsible for the continuing COVID-19 pandemic. Combining intramuscular and intranasal vaccination routes is a promising approach for achieving more potent immune responses. We evaluated the immunogenicity of prime-boost protocols with a chimpanzee adenovirus serotype 68 vector-based vaccine, ChAdTS-S, administered via both intranasal and intramuscular routes in BALB/c mice. Intramuscular priming followed by an intranasal booster elicited the highest levels of IgG, IgA, and pseudovirus neutralizing antibody titres among all the protocols tested at day 42 after prime immunization compared with the intranasal priming/intramuscular booster and prime-boost protocols using only one route. In addition, intramuscular priming followed by an intranasal booster induced high T-cell responses, measured using the IFN-γ ELISpot assay, that were similar to those observed upon intramuscular vaccination. All ChAdTS-S vaccination groups induced Th1-skewing of the T-cell response according to intracellular cytokine staining and Meso Scale Discovery cytokine profiling assays on day 56 after priming. This study provides reference data for assessing vaccination schemes of adenovirus-based COVID-19 vaccines with high immune efficacy.

## Introduction

Severe acute respiratory syndrome coronavirus-2 (SARS-CoV-2) caused the coronavirus disease 2019 (COVID-19) pandemic, threating global public health and safety [1]. According to the World Health Organization, as of March 20, 2022, over 468 million COVID-19 cases and almost 6 million related deaths had been reported globally [2]. Currently, variants of concern include Alpha (B.1.1.7), Beta (B.1.351), Delta (B.1.617.2), Gamma (P.1), and Omicron (B.1.1.529) [3, 4]. The protective efficacy of many COVID-19 vaccines against these new variants has been shown to decrease relative to that against the original Wuhan-Hu-1 strain [5–8]. Therefore, it is important to develop more effective vaccines, as well as immunization strategies, to improve vaccine efficacy.

Several adenovirus-based vaccines, including Ad5-nCoV (CanSinoBio), Sputnik V (Gamaleya Research Institute), and ChAdOx1 nCoV-19 (AZD-1222, Oxford/AstraZeneca), have been approved for emergency use. The protective efficacy of ChAdOx1 nCoV-19 (81%) against SRAS-CoV-2 was lower than that of the mRNA vaccine BNT162b2 (95%) [9, 10]. It is particularly concerning that the efficacy of adenovirus-based COVID-19 vaccines shows a decline against new variants [11]. Moreover, homologous prime-boost with Ad5-nCoV at a 56-day interval induces a limited immune response due to pre-existing anti-Ad5 immunity [12]. To overcome these restrictions, we urgently need a new immunization strategy to improve the efficacy of adenovirus-based COVID-19 vaccines.

Most COVID-19 vaccines are administered by intramuscular injection, which primarily induces an immunoglobulin G (IgG) response, but a low mucosal immune response [13–15]. Recent studies have shown that compared with intramuscular inoculation, intranasal inoculation of an adenovirus vaccine produced increased titres of neutralizing antibodies (NAbs) and increased protective efficacy [16, 17]. However, intranasal inoculation of adenovirus vector vaccines produces relatively poor cellular immune responses [18]; therefore, combining intramuscular and intranasal immunizations may overcome this bottleneck and increase immune efficacy.

The chimpanzee adenovirus serotype 68 vector (AdC68) has a low pre-existing immunity, showing a seroprevalence of only 10–15% in healthy Chinese adults [19]. Many AdC68-based vaccines exhibit potent immunogenicity and protective efficacy in mice [20, 21]. Here, we assessed the immunogenicity of a homologous prime-boost protocol using different immunization routes with the AdC68-based vaccine ChAdTS-S in BALB/c mice. This study provides reference data for further study of vaccination schemes for adenovirus-based COVID-19 vaccines to produce higher immune efficacy.

## Materials and methods

### Animals and vaccines

**Figure 1.**
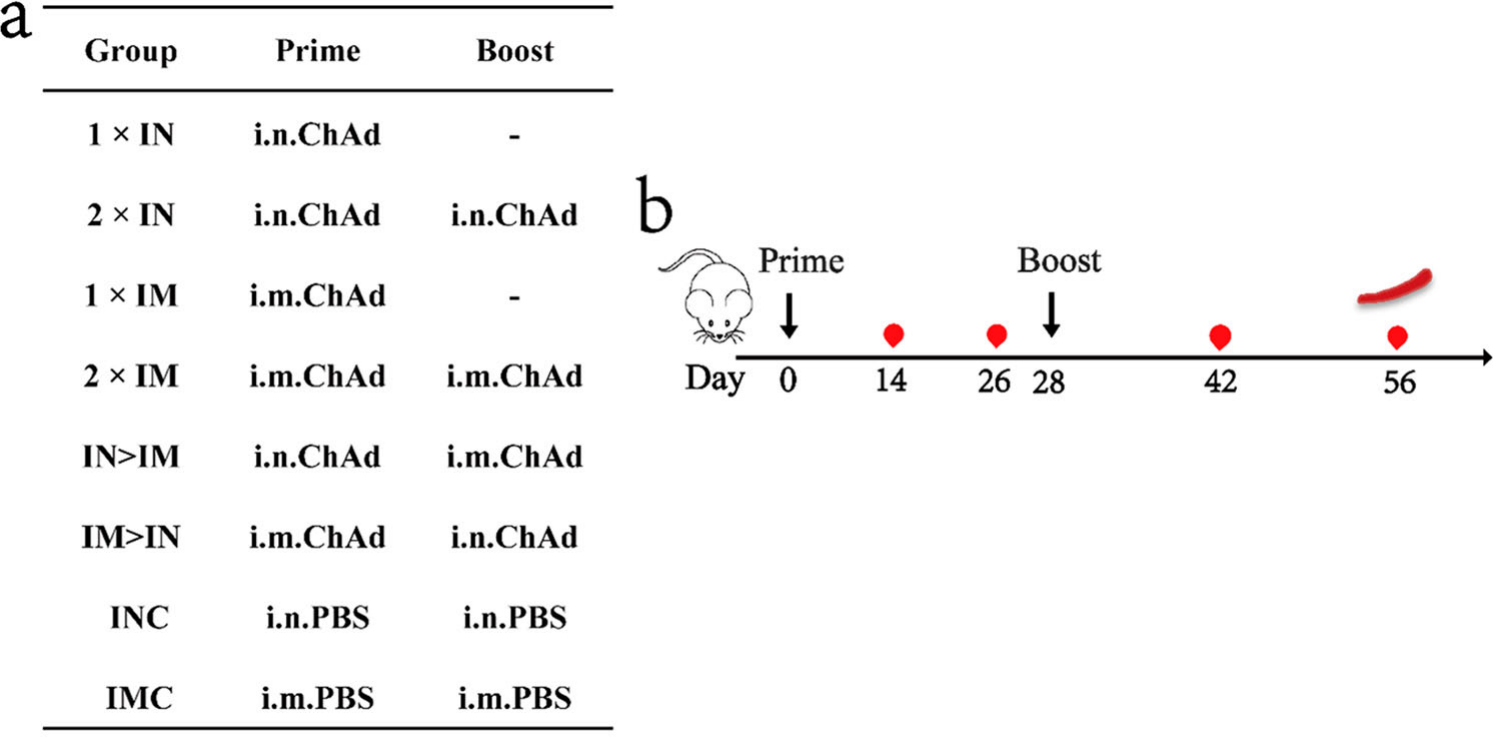
Overall scheme of the group design, immunization, and immunological characterization of female BALB/c mice. (a) Mice in 8 groups were immunized with ChAdTS-S via different immunization protocols. ChAd, Recombinant chimpanzee adenovirus vaccine ChAdTS-S; i.n., intranasal vaccination; i.m., intramuscular vaccination; (b) immunization and immunological characterization scheme. Dashes indicate no booster vaccination; indicates vaccination; indicates bleeding; indicates spleen lymphocyte isolation.

Experiments involving animals were approved by and carried out in accordance with the guidelines of the Institutional Experimental Animal Welfare and Ethics Committee of National Institutes for Food and Drug Control. Specific-pathogen-free female BALB/c mice, aged 6 weeks, were provided and housed by the National Institutes for Food and Drug Control. The vaccine used in this study was the chimpanzee adenovirus-vectored vaccine ChAdTS-S (1 × 10^11^ vp/0.5 mL, WALVAX Biotechnology Co., Ltd., Kunming, China). The mice were randomly divided into eight groups (n = 5) and immunized using different vaccine regimens: single-dose groups (1×IN, 1×IM), two-dose groups with the same vaccination route (2×IN, 2×IM), two-dose groups with different vaccination routes (IN > IM, IM > IN), blank control groups (INC, IMC). The overall scheme used for group design, immunization, and immunological characterization is shown in Figure 1a and b. The prime vaccination day was set as day 0, and the boost was performed on day 28. In total, 4 × 10^10^ vp of ChAdTS-S were used for each vaccination. Blood samples were collected from all mice on days 14, 26, 42, and 56. All experiments were independently performed twice.

### Enzyme-linked immunosorbent assay (ELISA)

SARS-CoV-2 spike protein and spike receptor-binding domain (RBD)-specific IgG and IgA titres were determined using ELISA. Briefly, Costar ELISA plates (Corning, Inc., Corning, NY, USA) were coated overnight with 0.2 μg SARS-CoV-2 spike protein or recombinant RBD protein (Sino Biological, Beijing, China). The plate was blocked with phosphate-buffered saline (PBS) containing 1% bovine serum albumin and 0.05% Tween 20 for 1 h at 37 °C. After washing the plates six times with PBS containing 0.05% Tween 20, sera were added to the wells at 4-fold serial dilutions. Plates were washed six times with PBS containing 0.05% Tween 20 and then incubated with horseradish peroxidase-conjugated goat anti-mouse IgG (ZSGB-BIO, Beijing, China, 1:10,000) or horseradish peroxidase-conjugated goat anti-mouse IgA (Abcam, Cambridge, UK, 1:10,000) for 1 h at 37 °C. After washing, 3,3’,5,5'-tetramethylbenzidine (TMB) (Beyotime, Shanghai, China) was used as the substrate to detect the antibody responses at 450 and 630 nm. The endpoint of the serum antibody titre was calculated as the reciprocal of the highest dilution, which was 2.1-fold higher than the optical absorbance value of the negative control.

### Recombinant VSV-based pseudovirus neutralization assay

Recombinant VSV-based pseudotyped SARS-CoV-2 was provided by the Division of HIV/AIDS and Sex-Transmitted Virus Vaccines, National Institutes for Food and Drug Control, including Wuhan-Hu-1 SARS-CoV-2, Delta (B.1.617.2) SARS-CoV-2, and Omicron (B.1.1.529) SARS-CoV-2. The assay was performed as previously described [22]. Briefly, mouse sera were inactivated in a water bath at 56 °C for 30 min. Three-fold serial dilutions of serum and 650 TCID_50_ (50% tissue culture infectious dose) of pseudovirus were mixed and incubated at 37 °C for 1 h. Vero cells (2 × 10^5^) were added and incubated at 37 °C with 5% CO_2_ for 24 h. The amount of pseudovirus that entered the target cells was calculated by detecting the expression of luciferase to measure NAbs. Relative luciferase activity was measured using a luciferase assay system (PerkinElmer, Waltham, MA, USA). A negative control containing only cells, and a virus control containing only virus and cells, were included in each plate. The half-maximal effective concentration was calculated for all tested samples. For neutralizing titres <30, we recorded the value as 30 for plotting.

### ELISpot assay

Mice were sacrificed and soaked in 75% ethanol. Spleens were removed in a 40 μm cell strainer with 4–5 ml mouse lymphocyte separation medium (Dakewe, Beijing, China) and ground with a 2 ml syringe piston. The suspension of spleen cells was immediately transferred to a 15 mL centrifuge tube, covered with 1000 μL RPMI 1640 medium (Hyclone, Cytiva, MA, USA), and centrifuged at 800 *g* for 30 min at room temperature. After centrifugation, the liquid in the 15 mL centrifuge tube was divided into four layers from the top to bottom: the RPMI 1640 medium covering layer, lymphocyte layer, separation fluid layer, and erythrocyte and cell fragment layer. The lymphocyte layer was sucked out; 10 mL RPMI 1640 medium was added. Lymphocytes were collected following centrifugation at 250 *g* for 10 min at room temperature. The supernatant was discarded, and the cells were suspended in serum-free medium (Dakewe). IFN-γ-positive cells were detected using a mouse IFN-γ ELISpot plus kit (Mabtech, Stockholm, Sweden). Briefly, wells of 96-well polyvinylidene fluoride plates were washed four times with 200 µL of PBS and blocked with RPMI-1640 medium containing 10% foetal bovine serum for at least 2 h at 24 °C. Freshly isolated lymphocytes (2.5 × 10^5^) were transferred to the wells and stimulated at 37 °C for 24 h with a peptide pool (1 µg/mL per peptide, Genscript, Nanjing, China) derived from a peptide scan (15-mers with 11-residue overlaps) of the entire spike glycoprotein of SARS-CoV-2. The plates were incubated with anti-mouse IFN-γ antibody at room temperature for 2 h and then with streptavidin-horseradish peroxidase (diluted at 1:1,000, Dakewe) for 1 h. After washing, 100 μL of TMB substrate solution was added per well and developed for 5 min until distinct spots emerged. Spots were imaged and counted using an ImmunoSpot S6 Universal instrument (Cellular Technology Limited, Shaker Heights, OH, USA).

### Intracellular cytokine staining

Splenic lymphocytes were isolated as described above, then stimulated for 6 h at 37 °C with 2 μg/mL of the spike protein peptide pool and brefeldin A (diluted at 1:1,000, Biolegend, San Diego, CA, USA) as described above to block cytokine secretion. Following stimulation, splenocytes were washed and stained with a mixture of the following antibodies against lineage markers: BV421 hamster anti-mouse CD3e antibody, BV510 rat anti-mouse CD4 antibody, and FITC rat anti-mouse CD8a antibody as well as the fixable viability stain 780 (all from BD Biosciences, San Jose, CA, USA) to exclude dead cells from analysis. After two washes with PBS, cells were fixed and permeabilized with Cytofix/Cytoperm (BD Biosciences), washed with Perm/Wash buffer (BD Biosciences), and stained with PE-conjugated rat anti-mouse IFN-γ, BV605 rat anti-mouse interleukin (IL)−2, PE-Cy7 rat anti-mouse IL-4, APC rat anti-mouse IL-10, and BB700 rat anti-mouse tumour necrosis factor (TNF) (all from BD Biosciences). Cells were washed successively with Perm/Wash buffer and PBS, then resuspended in PBS and subjected to flow cytometry using a FACS Lyric analyser (BD Biosciences). At least 200,000 events were collected for each sample. Data were analysed using FlowJo software (TreeStar, Ashland, OR, USA). CD8^+^ and CD4^+^ T cells were gated from single cells (FSC-A vs. FSC-H), lymphocytes (FSC-A vs. SSC-A), and live CD3^+^ T cells (CD3^+^ vs. LD780^−^); detection data are presented as percentages of cytokine-positive cells among CD8^+^ or CD4^+^ T cells.

### Meso Scale Discovery (MSD) Th1/Th2 cytokine profiling

Supernatants were collected from ELISpot plates and assayed for TNF-α, IL-2, IL-4, IL-5, IL-6, and IL-10 using a V-PLEX Proinflammatory Panel 1 (mouse) Kit (Meso Scale Diagnostics, Rockville, MD, USA). Cytokine levels were determined using a MESOTM QuickPlex SQ 120 (Meso Scale Diagnostics). Concentrations were calculated using a standard curve.

### Statistical analysis

GraphPad Prism 9.0 software (GraphPad, Inc., San Diego, CA, USA) was used for analysis and data plotting. Data are presented as geometric means ± geometric standard deviation. One-way analysis of variance was used to detect statistical significance among groups (*P < 0.05; **P < 0.01; ***P < 0.001; ****P < 0.0001; ns, no significance).

## Results

### Homologous prime-boost with ChAdTS-S by combining intramuscular and intranasal routes elicits a potent humoral immune response in mice

**Figure 2.**
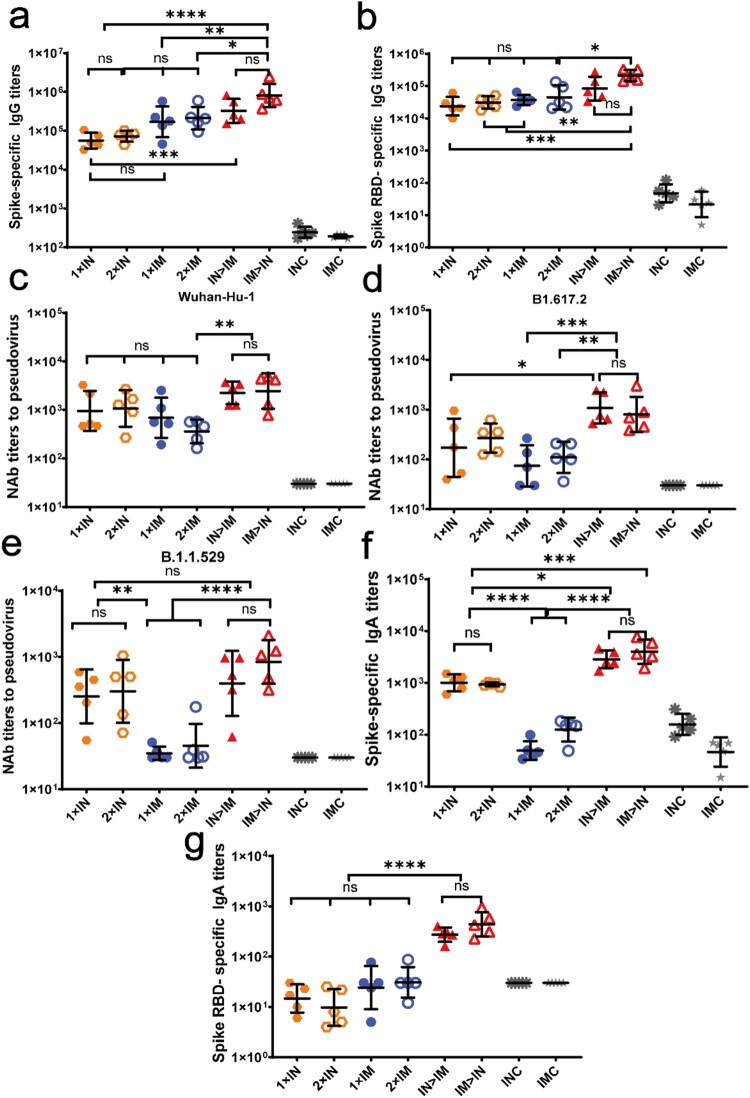
Humoral immune responses induced by ChAdTS-S vaccine using different vaccination protocols. All titres were measured at day 42 after prime immunization. **(a)** Serum spike-specific binding IgG titres (n = 5 per group, each spot represents one sample). **(b)** Serum spike RBD-specific binding IgG titres (n = 5 per group, one spot represents one sample). **(c-e)** Serum NAb titres against **(c)** Wuhan-Hu-1, **(d)** B.1.617.2, and **(e)** B.1.1.529. NAb titres are expressed as 50% inhibitory dilutions (n = 5 per group, one spot represents one sample). **(f)** Serum spike-specific IgA binding titres (n = 5 per group, each spot represents one sample). **(g)** Serum spike RBD-specific IgA binding titres (n = 5 per group, one spot represents one sample). Bars represent geometric means ± geometric SD; *P < 0.05; **P < 0.01; ***P < 0.001; ****P < 0.0001; ns: *P* > 0.05.

The prime-boost designs are outlined in Figure 1a and b. Spike-specific and RBD-specific IgG titres in serum were detected using ELISA on day 42 after primary immunization to evaluate the systemic immune response. As shown in Figure 2a, the intramuscularly prime and intranasally boosted group (IM > IN) developed the highest levels of spike-specific IgG antibody titres among all tested groups. The IgG geometric mean titre (GMT) in group IM > IN was significantly higher than those in groups 1 × IN (*P* < 0.0001), 2 × IN (*P* < 0.0001), 1 × IM (*P* = 0.004), and 2 × IM (*P* = 0.0187), showing increases of 13.5-, 10.2-, 3.8, 2.8-fold, respectively. The IgG GMT in group IM > IN was 2.5-fold of that in the ChAdTS-S intranasal prime and intramuscularly boosted group (IN > IM), but the IgG titres between these two groups showed no significant differences (*P* = 0.2418). A second dose, administered either intranasally or intramuscularly via the same route, did not significantly elevate spike-specific IgG responses over those with only one dose.

Similar to the data for the spike-specific IgG antibody, IM > IN produced the highest RBD response (Figure 2b). The RBD-specific IgG GMT of IM > IN was significantly higher than those of groups 1 × IN (*P* = 0.0004), 2 × IN (*P* = 0.0022), 1 × IM (*P* = 0.0064), and 2 × IM (*P* = 0.0204), with increases of 32.9-, 24.9-, 20.9-, and 17.0-fold, respectively. Compared with that of IN > IM, the RBD-specific IgG GMT of IM > IN increased by 8.6-fold. Nevertheless, there was no significant difference between the RBD-specific IgG GMTs of IM > IN and IN > IM (*P* = 0.4025). A second dose, administered either intranasally or intramuscularly via the same route, did not significantly elevate RBD responses over those produced by a single dose.

NAb titres may be highly correlated with vaccine efficacy [23]. Virus-specific serum NAb titres against the Wuhan-Hu-1 strain, B.1.617.2 variant, and B.1.1.529 variant were assessed using VSV-based pseudovirus assays at day 42 after primary immunization (Figure 2c, d, e). In response to the Wuhan-Hu-1 pseudovirus (Figure 2c), the NAb GMTs of groups IM > IN and IN > IM were higher (2451 and 2242, respectively) than those of the other groups; the NAb GMTs of groups IM > IN and IN > IM were not significantly different (*P* > 0.9999). The remaining four ChAdTS-S vaccination groups produced relatively low NAb GMTs, with values of 953 in group 1 × IN, 1071 in group 2 × IN, 689 in group 1 × IM, and 360 in group 2 × IM, which were not significantly different. Higher NAb GMTs were obtained after both one and two intranasal doses than those obtained after one and two intramuscular vaccinations. Virus-specific NAbs were not detected in the IMC and INC groups.

NAb titres against B.1.617.2 pseudovirus were reduced compared to those against the Wuhan-Hu-1 pseudovirus (Figure 2d) in all ChAdTS-S vaccinated groups. The NAb GMTs against B.1.617.2 pseudovirus of groups IM > IN and IN > IM were the highest among all groups, with GMTs of 804 and 1083, respectively, which were not significantly different (*P* = 0.9986). The NAb GMTs of groups IM > IN and IN > IM against B.1.617.2 pseudovirus decreased by 2.1- and 1.1-fold, respectively, compared to those against Wuhan-Hu-1 pseudovirus. The NAb GMTs of groups 1 × IN, 2 × IN, 1 × IM, and 2 × IM against B.1.617.2 pseudovirus were 172, 268, 74, and 110, respectively, showing a decrease of 4.5-, 3.0-, 8.3-, and 2.3-fold, respectively, relative to those against Wuhan-Hu-1 pseudovirus.

Virus-specific NAb titres against the B.1.1.529 pseudovirus were reduced compared to those against Wuhan-Hu-1 pseudovirus in all ChAdTS-S vaccinated groups (Figure 2e). The NAb GMT of group IM > IN against B.1.1.529 pseudovirus was 840, which was the highest among all groups. The remaining ChAdTS-S groups produced relatively low NAb GMTs against B.1.617.2 pseudovirus: those of 1 × IN, 2 × IN, 1 × IM, 2 × IM, and IN > IM were 254, 302, 35, 45 and 397, respectively, showing a decrease of 2.8-, 2.5-, 18.8-, 7.0-, 4.6-fold, respectively, relative to those against Wuhan-Hu-1 pseudovirus.

Mucosal immunity was assessed by measuring spike- and RBD-specific serum IgA titres using ELISA on day 42 after prime immunization (Figure 2f, g). Spike-specific ELISA (Figure 2f) showed that all four intranasal groups exhibited high spike-specific IgA titres on day 42 after prime immunization, with GMTs of 4003 in IM > IN, 2851 in IN > IM, 1005 in 1 × IN, and 931 in 2 × IN. Groups IM > IN and IN > IM showed the highest IgA titres, with no significant differences between these two groups (*P* = 0.9354). The IgA GMT of group IM > IN was significantly higher than those in groups 1 × IN (*P* < 0.001) and 2 × IN (*P* < 0.0005), showing increases of 3- and 3.2-fold, respectively. The IgA GMTs of 1 × IM and 2 × IM were 49 and 126, respectively. As expected, intramuscular vaccination did not elicit significant mucosal immune responses, with IgA titres similar to those of groups IMC and INC.

Using RBD-binding IgA ELISA (Figure 2g), groups IM > IN and IN > IM induced the highest titres, with GMTs of 440 and 273, respectively; these were significantly higher than those of the 1 × IN, 2 × IN, 1 × IM, and 2 × IM groups (*P* < 0.0001). However, groups IM > IN and IN > IM did not significantly differ in RBD-specific IgA titres (*P* = 0.9164).

**Figure 3.**
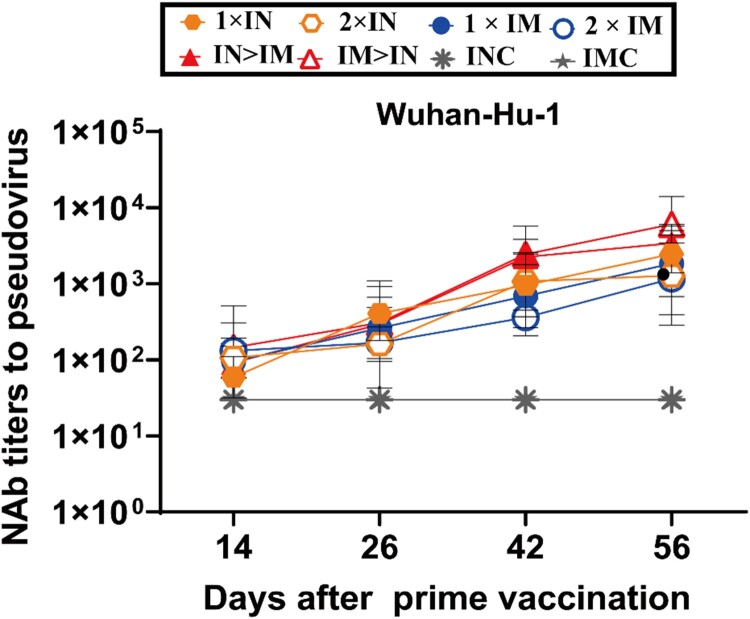
Serum NAb titre levels were assessed based on the Wuhan-Hu-1 strain SARS-CoV-2 pseudovirus. Serum samples were collected on days 14, 26, 42, and 56 after prime vaccination; NAb titres are expressed as 50% inhibitory dilutions (n = 5 per group, one spot represents GMT per group at each time point). Bars represent geometric means ± geometric SD; *P < 0.05; **P < 0.01; ***P < 0.001; ****P < 0.0001; ns: *P* > 0.05.

Serum samples were collected on days 14, 26, 42, and 56 after primary vaccination to evaluate the rising tendency of neutralizing activity against Wuhan-Hu-1 (Figure 3). The NAb GMTs of all ChAdTS-S vaccinated groups continued to increase throughout the 8 weeks after primary vaccination.

### All ChAdTS-S vaccinated groups exhibit strong T-cell responses

**Figure 4.**
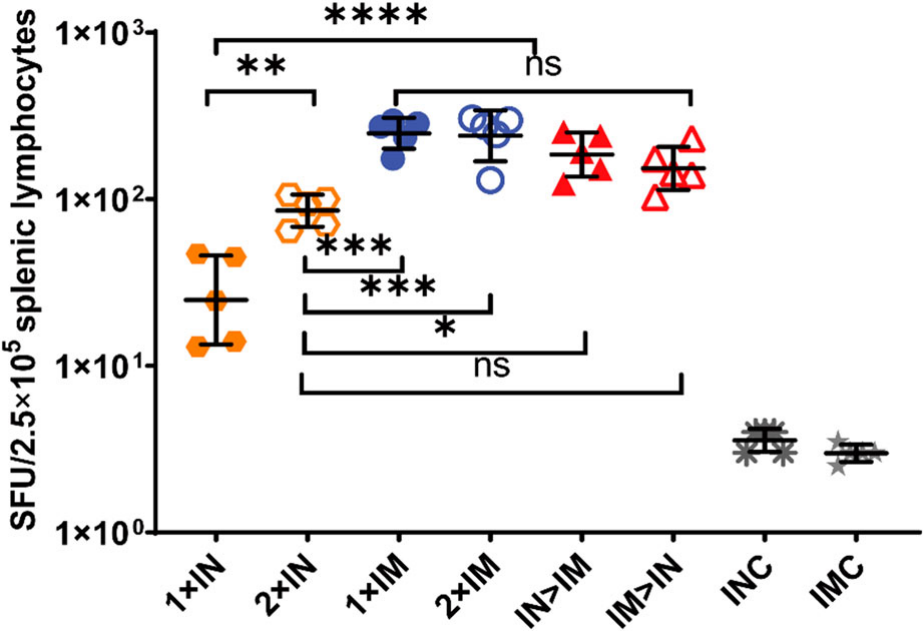
SARS-CoV-2 spike protein-specific cellular immune responses following ChAdTS-S vaccination. SARS-CoV-2 spike-specific IFN-γ detected using enzyme-linked immunospot assays. Five mice from each group were euthanized and their T-cell responses were measured. Lymphocytes were stimulated with SARS-CoV-2 spike peptide pools spanning the entire spike protein. IFN-γ-secreting cells were quantified using an ELISPOT assay (n = 5 per group; each data point represents the mean number of spots from double wells for one sample). Bars represent geometric means ± geometric SD, *P < 0.05; **P < 0.01; ***P < 0.001; ****P < 0.0001; ns: *P* > 0.05.

Splenic lymphocytes were collected on day 56 after primary immunization and stimulated with peptide pools spanning the SARS-CoV-2 spike protein for 24 h, after which IFN-γ ELISpot assays were performed (Figure 4). All ChAdTS-S vaccinated groups exhibited strong T-cell responses. Groups IM > IN, IN > IM, 1 × IM, and 2 × IM showed 153, 185, 248, and 240 spot-forming units per 2.5 × 10^5^ splenic lymphocytes, respectively, with no significant differences among these four groups. The 1 × IM protocol elicited higher spike-specific T-cell responses than 1 × IN (*P* < 0.0001), and the 2 × IM protocol induced higher spike-specific T-cell responses than the 2 × IN protocol (*P* = 0.0003). Interestingly, a second intranasal dose significantly elevated the T-cell responses over those of single intranasal dose (*P* = 0.0016), with geometric means of 85 and 25, respectively.

### ChAdTS-S vaccines induce Th1-skewing of the T-cell response

We further investigated Th1 skewing of spike protein-specific T-cell responses. Splenic lymphocytes were collected at day 56 after prime immunization and stimulated with an overlapping spike protein peptide pool, and intracellular cytokine staining and MSD cytokine profiling assays were conducted to assess Th1-dominant T-cell responses.

**Figure 5.**
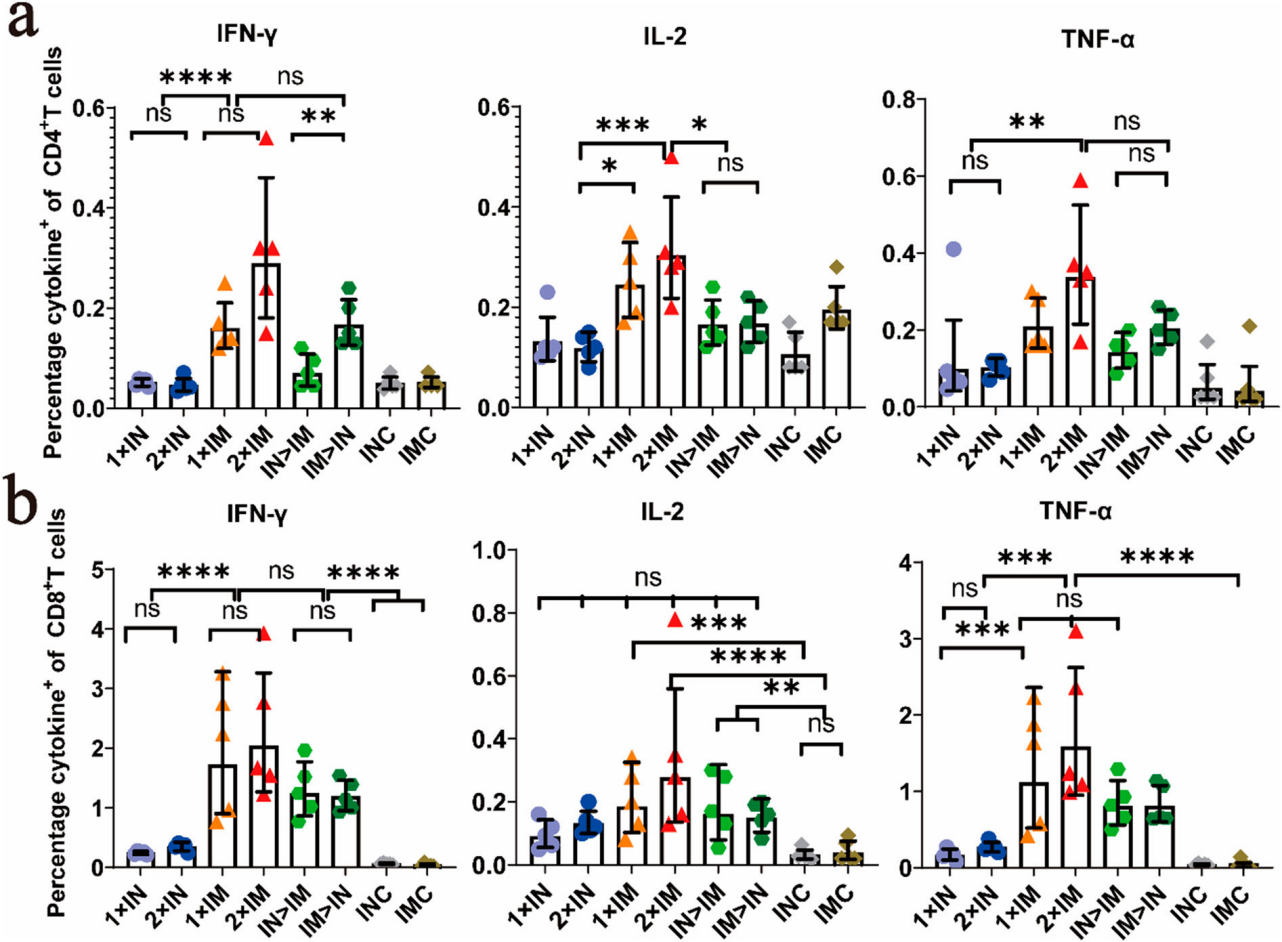
Th1/Th2 skewing detected by intracellular cytokine staining in ChAdTS-S immunized mice. Lymphocytes were stimulated with SARS-CoV-2 spike peptide pools spanning the entire spike protein for 8 h. Percentage of spike protein-specific IFN-γ, IL-2, TNF-α positive memory CD4^+^ T (a) and CD8^+^ T (b) cells, measured at day 56 after prime immunization (n = 5 per group, one spot represents one sample). Bars represent the geometric means ± geometric SD, *P < 0.05; **P < 0.01; ***P < 0.001; ****P < 0.0001; ns: *P* > 0.05.

Intracellular cytokine staining showed a high percentage of splenic lymphocytes positive for hallmark Th1 cytokines IFN-γ, TNF-α, and IL-2 in all ChAdTS-S-vaccinated groups, but a low percentage of secreted typical cytokines (IL-4 and IL-10) associated with the Th2-type immune response (Figure 5a, b). The proportions of splenic lymphocytes secreting cytokines IL-4 and IL-10 in CD4^+^ and CD8^+^ T cells are provided in the Supplementary materials (S1a, b). There was a higher Th1 response in the intramuscular vaccination (i.m.) group than in the intranasal vaccination (i.n.) group. Groups IM > IN and IN > IM showed Th1 responses similar to those of groups 1 × IM and 2 × IM.

**Figure 6.**
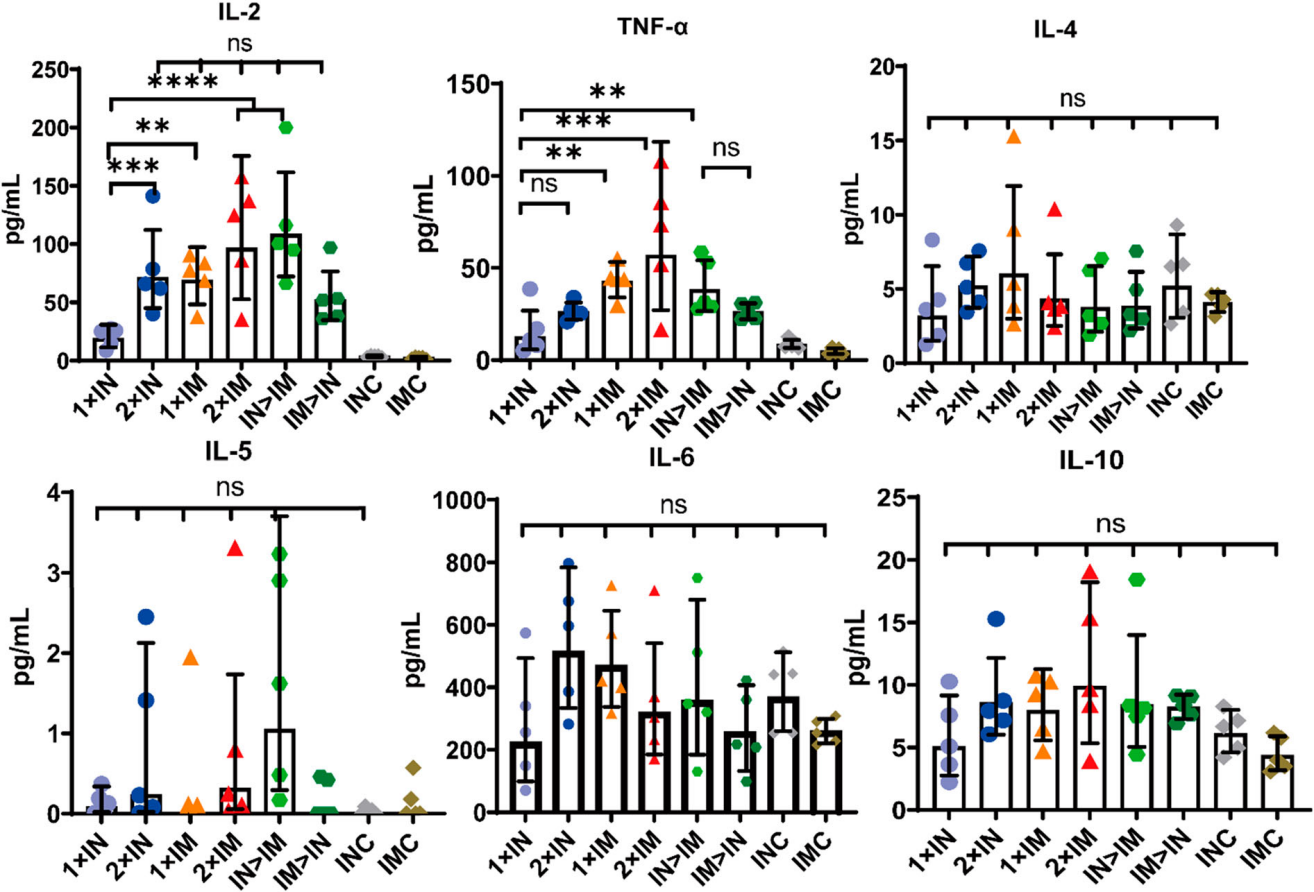
Th1/Th2 skewing in ChAdTS-S immunized mice measured using MSD cytokine profiling. Lymphocytes were stimulated with SARS-CoV-2 spike peptide pools spanning the entire spike protein for 24 h. IL-2, TNF-α, IL-4, IL-5, IL-6, and IL-10 levels in supernatants were measured (n = 5 per group, one spot represents one sample). Bars represent geometric means ± geometric SD, *P < 0.05; **P < 0.01; ***P < 0.001; ****P < 0.0001; ns: *P* > 0.05.

MSD cytokine profiling assays for TNF-α, IL-2, IL-4, IL-5, IL-6, and IL-10 were performed to assess the function and polarization of spike protein-specific T cells (Figure 6). All ChAdTS-S-vaccinated groups elicited high TNF-α and IL-2 concentrations, with a higher level in the intramuscular group than in the intranasal group, and low concentrations of IL-4, IL-5, and IL-10 were detected. Furthermore, there was no significant difference in IL-6 concentration among all the groups, including IMC and INC groups. These results suggest that Th1-skewed response increased significantly in all ChAdTS-S-vaccinated groups, and that Th2-skewed response did not increase.

## Discussion

Many SARS-CoV-2 variants show immune evasion in response to some COVID-19 vaccines [24–26]. Developing new vaccination strategies with adenovirus vector-based COVID-19 vaccines against SARS-CoV-2 variants is imperative for improving vaccine efficacy. Several studies have shown that intranasal administration of adenovirus vector-based COVID-19 vaccines induced higher NAb levels, IgA responses and protective efficacy against SARS-CoV-2, but lower cellular immune responses, compared to intramuscular administration [18, 27, 28]. In our investigation, intramuscular prime and intranasal boost regimen of ChAdTS-S not only elicited the highest levels of IgG, IgA, and NAb titres, but also induced high cellular immune responses comparable to intramuscular vaccination. Thus, it might be a promising immunization strategy for adenovirus vector-based vaccines. We hypothesized that intranasal boost vaccination of ChAdTS-S could bypass anti-vector immune responses induced by the intramuscular prime vaccination, leading to significant increases in systemic and local mucosal immune responses. Further research is needed to determine whether this is true. Interestingly, Croyle.et al [29] found in the presence of anti-Ad5 pre-existing immunity, mice inoculated intranasally with Ad5 vector expressing Ebola Zaire glycoprotein produced potent systemic immune responses and complete protection against Ebola virus, while mice inoculated intramuscularly and orally did not.

Increasing the inoculation interval of adenovirus vector-based vaccine may produce higher antibody titres. Solforosi et al. [30] studied the immunogenicity and efficacy of two doses of the Ad26.COV2. S COVID-19 vaccine and found that the NAb titres produced with an 8-week interval were higher than those produced with 4-week intervals. The efficacy of AZD1222 tended to be higher when the interval between the two intramuscular doses was more than 12 weeks than when it was less than 6 weeks in subjects aged 18–55 years [31]. Wu et al. [32] evaluated the safety, tolerability, and immunogenicity of aerosolized Ad5-nCoV administered via nebulization inhalation at day 28 after primary immunization. The two-dose aerosolized Ad5-nCoV was well-tolerated and had few side-effects. Ad5-nCoV administered intramuscularly combined with aerosol inhalation induced the highest IgG, IgA, and NAb titres and high cellular immune responses at day 56 after primary immunization, which is consistent with our results. Intramuscular prime and intranasal boost vaccination could elicit high immune responses in shorter time than the prime-boost vaccination via the same route.

Intranasal inoculation of ChAdTS-S was used to induce rapid secretory IgA production in memory B cells by simulating the natural infection process of SARS-CoV-2 [33]; this is more likely to protect the upper respiratory tract from SARS-CoV-2 infection, resisting the virus at its point of entry [28]. We found that homologous prime-boost with ChAdTS-S administered intramuscularly combined with intranasal vaccination induced higher spike-specific and RBD-specific IgA responses at day 42 after primary immunization than any other protocol did. Potent mucosal immune response may provide early and rapid neutralizing antibody response to new SARS-CoV-2 variants [34]. Many studies have suggested that the increased efficacy is related to the strong neutralizing activity of IgA against SARS-CoV-2 in the early stage of infection [35, 36]. For example, nasal spray influenza vaccines confer protection similar to intramuscular injection, although with a decreased humoral immune response [37, 38]. Intranasal vaccination with ChAdOx1 nCoV-19/AZD1222 protected rhesus macaques against pneumonia and suppressed shedding of SARS-CoV-2 harbouring the D614G substitution in the spike protein, whereas intramuscular injection did not [39].

The T-cell response plays an important protective role in recognizing and killing infected cells and secreting specific antiviral cytokines [40]. Furthermore, T-cell responses are also necessary for generating humoral immune responses to SARS-CoV-2 in mice [41]. Therefore, a robust cellular immune response is critical to induce early COVID-19 protection and amplify subsequent immune response. Here, all ChAdTS-S vaccination groups induced Th1-skewing of the T-cell response on day 56 after prime immunization, similar to responses in mRNA-based and adenovirus vector-based COVID-19 vaccines [42, 43]. We found that intramuscular prime and intranasal boost vaccination with ChAdTS-S, which was equivalent to that of the intramuscular administration, induced a stronger Th1 immune response than did intranasal administration alone; this might reduce the risk of vaccine-enhanced disease.

Our study has several limitations. First, we did not measure neutralization titres for live SARS-CoV-2 because of limited resources. NAb levels are strongly associated with immune protection against symptomatic SARS-CoV-2 infection [22], and further studies are needed to confirm the efficacy of the IM > IN route using SARS-CoV-2 challenge. Second, although the IgG and NAb GMTs between groups IM > IN and IN > IM differed, the differences were not statistically significant. This result requires validation with a larger sample size. Third, we did not analyse secretory IgA responses in the nasopharynx that may be particularly important in protection against SARS-CoV-2 infection [34].

In conclusion, combining homologous intramuscular prime and intranasal boost vaccination with ChAdTS-S induced a stronger and broader immune response, indicating a promising strategy against SARS-CoV-2, particularly with respect to emerging variants. This strategy should be further examined in clinical trials.

## Supplementary Material

Supplemental Material

## Data Availability

All data supporting this study are presented in the paper. The original datasets are also available from the corresponding author upon request.
