# Supplementary material for: Combining intramuscular and intranasal homologous prime-boost with a chimpanzee adenovirus-based COVID-19 vaccine elicits potent humoral and cellular immune responses in mice
Source: Emerg Microbes Infect. 2022 Jul 27;11(1):1890–9. doi: 10.1080/22221751.2022.2097479 (PMC9331206; doi:10.1080/22221751.2022.2097479)
Supplement: Supplemental Material [file TEMI_A_2097479_SM5112.zip › Supplementary Materials.docx]

**Supplementary Appendix**

**Figure S1**


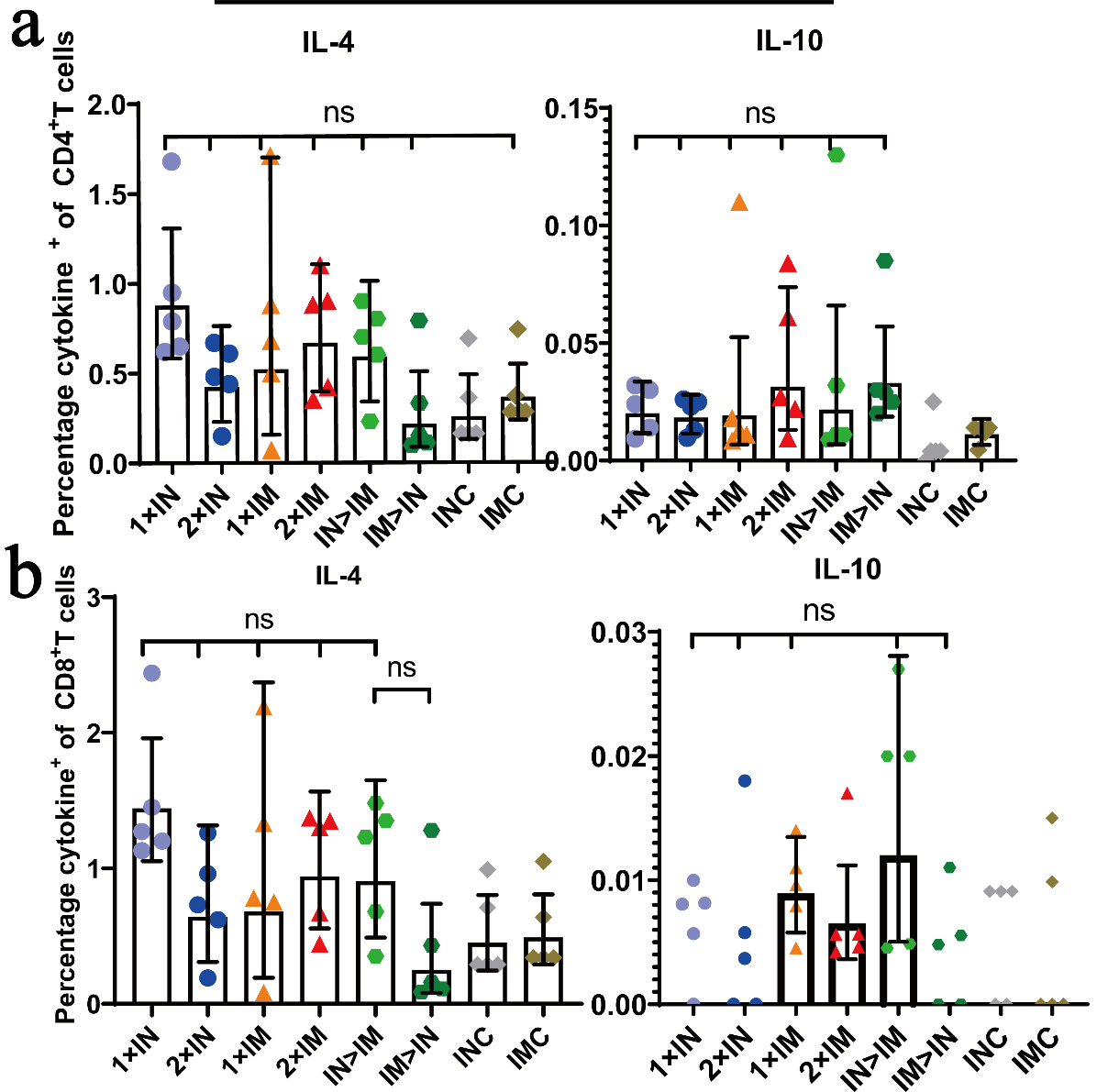


Figure S1. Th1/Th2 skewing in ChAdTS-S immunized mice. Spike protein-specific IL-4 and IL-10 responses in memory CD4^+^ T (a) and CD8^+^ T (b) cells, measured at day 56 after prime immunization. Responses were detected by intracellular cytokine staining (n = 5 per group; one spot represents one sample). Bars represent the geometric means ± geometric SD, *P < 0.05; **P < 0.01; ***P < 0.001; ****P < 0.0001; ns: P > 0.05.
