# Supplementary figures and images for: Combining intramuscular and intranasal homologous prime-boost with a chimpanzee adenovirus-based COVID-19 vaccine elicits potent humoral and cellular immune responses in mice
Source: Emerg Microbes Infect. 2022 Jul 27;11(1):1890–9. doi: 10.1080/22221751.2022.2097479 (PMC9331206; doi:10.1080/22221751.2022.2097479)

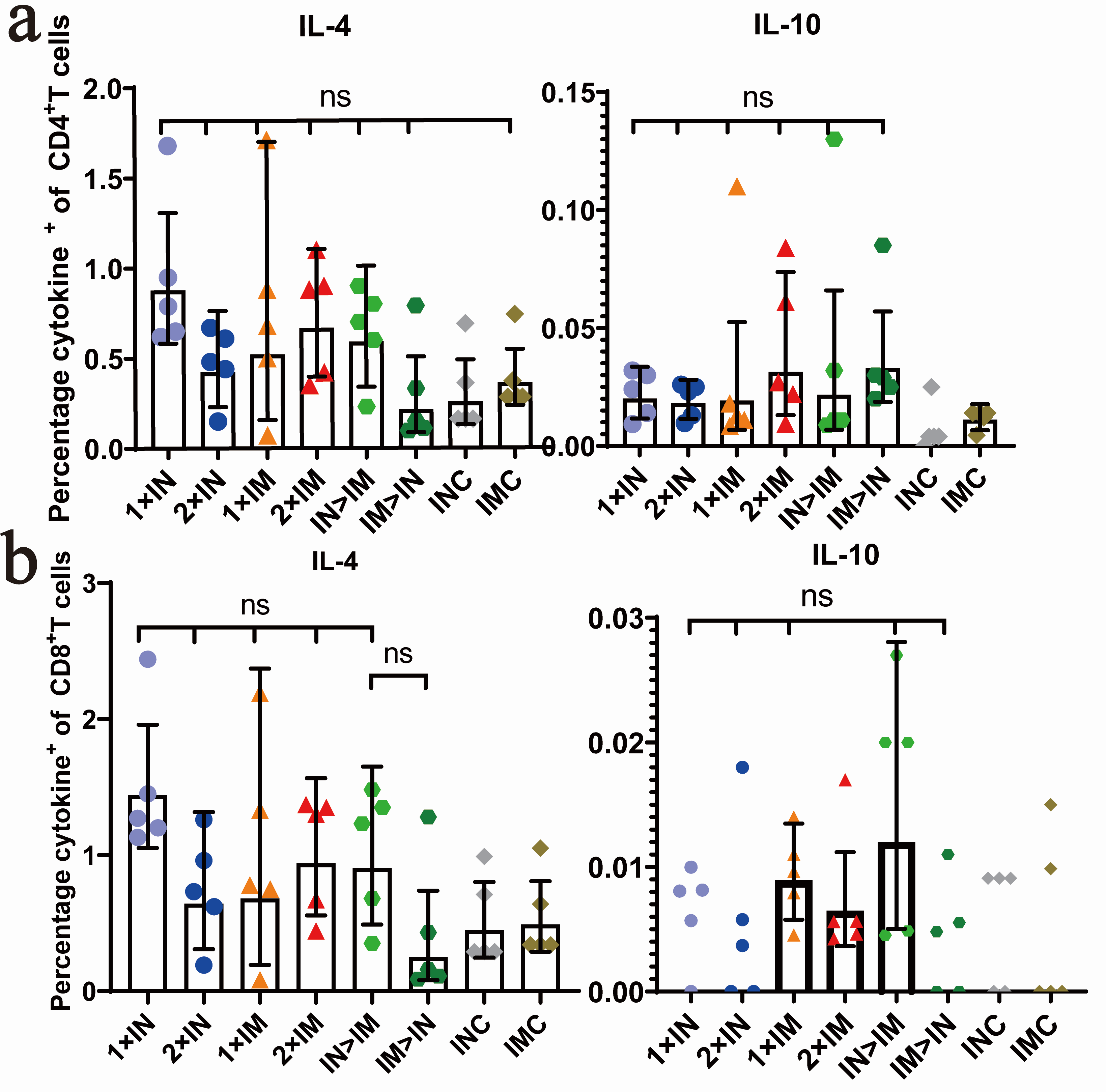

Supplement: Supplemental Material [file TEMI_A_2097479_SM5112.zip › Figure_S1.tiff]
